# Intellectual Property Rights and Global Access to Health Technologies During Pandemics: Reflecting on Vaccine Nationalism, COVID-19 & the WHO Pandemic Agreement Negotiations — The Need for Collective Action and Institutional Change

**DOI:** 10.1017/jme.2025.10149

**Published:** 2025

**Authors:** Aisling M. McMahon

**Affiliations:** School of Law and Criminology, Maynooth University, Ireland

**Keywords:** patents, Pandemic Agreement, access to health, vaccine nationalism, licensing conditions

## Abstract

Focusing on intellectual property rights (IPRs) and their role in global access to vaccines during the COVID-19 pandemic, this article argues that key aspects of the current institutional system align towards delivering individualistic state/regional/rightsholders priorities in the use of IPRs over pandemic health technologies. This played a key role in the vaccine nationalism and global vaccine inequity that emerged during the pandemic. It critically analyzes the IPR provisions within the World Health Organisation’s Pandemic Agreement and negotiation process. It argues that nationalistic/individualistic approaches toward the use of IPRs over health technologies also permeate such contexts. The final text of the Agreement leaves considerable discretion to states around IPRs, and much will depend on how it is implemented in practice. For effective future pandemic preparedness around how IPRs are used over health technologies, this article argues that a deeper bottom-up institutional change is needed — one which offers nuanced strategies to balance the potential incentivization role of IPRs with the implications certain uses of IPRs can have on access to downstream health technologies. A key element of this change is embedding a greater recognition of the range of resources provided by entities (e.g. funders, biobanks, and universities) necessary in the successful development of health technologies, including in pandemic contexts. Such entities should leverage these resources, including by attaching contractual conditions to access these, which mandate avenues for downstream access to pandemic health technologies. In the longer term such approaches could be part of a broader institutional change, which prioritises global collective health needs in pandemics.

## Introduction

*“No man is an island*,

*Entire of itself;*

*Every man is a piece of the continent*,

*A part of the main…”*

John Donne, Excerpt from “No Man is an Island”^1^

Pandemics by their nature affect many people.^2^ Given the scale and spread of disease, pandemics can bring not just significant global health impacts but also global economic and societal impacts.^3^ It is trite to say that infectious disease knows no, nor does it respect any, geographical boundaries of state or region.^4^ Yet, despite the global nature of such threats, legal responses to delivering access to health technologies that are needed to tackle health emergencies, including pandemics, often tend to be individualistic state-based or regional (for example, EU) responses which in many cases prioritize national or regional needs.^5^

For instance, the development and delivery of effective health technologies during the COVID-19 pandemic, including diagnostics, medicines, and vaccines, was key to the pandemic response. Yet, during the pandemic, once such technologies were developed, in many cases, there were major limitations around developing global pathways and coordination of these towards the rapid upscaling of production and distribution of such health technologies to meet global demand. In particular, a significant inequity developed between high income countries (HICs) and low- and- middle income countries (LMICs) in their access to COVID-19 vaccines.^6^ At the height of the COVID-19 pandemic, LMICs fell vastly behind HICs in their ability to secure access to COVID-19 vaccines to meet their populations’ needs.^7^ At the same time, HICs and regions such as the EU made advance purchase agreements to secure access to COVID-19 vaccine doses for their populations, in many cases securing enough agreed vaccine doses to ensure several doses per person in their populations.^8^ HICs and regions such as the EU tended to prioritize, first and foremost, their own state or regional needs, and in the vaccine context this became termed as vaccine nationalism.^9^

In practice, intellectual property rights (IPRs) played a key role in how vaccines and other health technologies were distributed to states during the COVID-19 pandemic, as a range of IPRs applied over different elements of health technologies. Given the exclusive nature of IPRs (discussed below), they enabled rightsholders to govern who could obtain access first to such health technologies and on what terms.^10^ Such rightsholders often tend to be private companies driven by a legal duty towards shareholders’ needs focusing in many cases primarily on maximizing potential profits.^11^ Whilst all states scrambled to obtain access to vaccines, HICs, having more access to resources and greater leverage, obtained access to such vaccines first ahead of LMICs in many cases.

Taking COVID-19 as a case study and focusing on IPRs, which were a key factor that impacted the development, distribution, and access to health technologies during the COVID-19 pandemic,^12^ this article argues that key aspects of the institutional framework including legal and political aspects of the system aligned towards delivering individualistic state/regional/rightsholder’s priorities. This in turn also played a key role around why broader collective health needs such as achieving global equitable access to such health technologies became marginalized.

Furthermore, this article argues that even after the pandemic, such nationalistic or in other ways individualistic approaches towards the use of IPRs over health technologies have (often) remained. This poses significant risks for future pandemic preparedness and response. Reflecting on recent international negotiations and discussions around the World Health Organization’s Pandemic Agreement,^13^ it will highlight significant difficulties in gaining consensus during this process on legally binding provisions on IPRs and technology transfer, including those related to equitable access to health technologies in pandemic contexts. It will demonstrate that many of the provisions related to IPRs in the recently adopted Pandemic Agreement (May 2025)^14^ are a significantly watered-down version of the original proposals. Whilst a change of provisions between the initial and final text of an agreement is common in international lawmaking, the article argues the changes over IPRs are symbolic of an individualistic approach to IPRs, which simply does not align with what is needed in pandemic contexts.

Having said that, the WHO Pandemic Agreement (May 2025) makes several references to the need for global equity and has a range of provisions that could be interpreted in ways that facilitate actionable pathways towards this. Much will depend on how this instrument is implemented in practice. For such provisions to have benefits in practice a cultural shift is needed, one which focuses on a deeper bottom-up change of the health innovation landscape that recognizes (and prioritizes) the need for global collective action in such contexts towards the rapid scale-up and sharing of health technologies, including addressing any IP obstacles to this.

Arguably, a key element of such a cultural shift is the need for greater recognition of the range of contributions needed to develop pandemic health technologies, alongside the inventors (and/or rightsholders) contributions. Moreover, this article argues that there should be greater leveraging of the contributions of other resource providers (such as funders, biobanks, universities, etc.) necessary for the development of pandemic health technologies, through encouraging such providers to attach “ethical access” licensing conditions (via contractual provisions) on the provision of such resources. In such contexts, access to their resources would attach conditions, including conditions related to delivering equitable access, to health technologies developed using such resources,^15^ particularly during pandemics. Such a change could be used to normalize the role of other actors (aside from rightsholders) exercising discretion and a say over how IPRs should be used, particularly in pandemic contexts. It would provide alternative avenues towards facilitating access to such health technologies.

In taking this focus, the article makes three main arguments: (1) That individualistic approaches to the use of IPRs over health technologies, and state/regional approaches to the procurement of health technologies, are often institutionally embedded within legal and political systems. This can make top-down change such as via the adoption of international agreements on IPR sharing, or the implementation of these provisions in ways that prioritize collective global needs, difficult to achieve without deeper institutional change. However, in a pandemic context where global access to health technologies is needed to tackle a virus, such individualistic modes of thinking can exacerbate health risks not just for individuals, states, or even regions, but for humanity more generally. (2) To ensure greater equitable access to pandemic health technologies, it will argue that alongside seeking to impose top-down legal provisions on IP sharing such as via an international Pandemic Agreement, deeper institutional change is needed, which requires a bottom-up approach. Such an approach is needed to embed a system that recognizes the need for global equitable access to such technologies, particularly during pandemics, and which in turn ensures strategies to achieve this are normalized across the health innovation system.^16^ (3) It makes the case that one key element to achieving this is for there to be a greater recognition of the range of resources and hence other resource providers (aside from the work of inventors/rightsholders) needed in developing effective health technologies, such as research funders, universities, biobanks, etc. Such actors can and should leverage access to their resources in ways that mandate greater accessibility of the downstream health technologies developed, particularly in pandemic contexts, including via the adoption of “ethical access licensing clauses.”^17^ Yet, for such approaches to be successful, it will argue that collective action is needed by these resource providers to leverage effective change.

The article is structured as follows: Part I examines the COVID-19 context as a case study to demonstrate how IPRs were used in ways that impacted access and distribution of pandemic health technologies and that IPRs were a key factor contributing to the global vaccine inequity during the pandemic. It then draws on scientific evidence to highlight the significant health implications of this individualized approach to IPRs during pandemic contexts, and to demonstrate the need for collective action towards delivering on global health needs for future pandemics. However, Part II argues that such individualized modes of thinking are also evident in the international negotiations around the recently adopted WHO Pandemic Agreement (May 2025). It was difficult to gain agreement on provisions on IPRs during the drafting of the WHO Pandemic Agreement, and the provisions on IPRs that were adopted are (in many cases) considerably watered-down versions when compared with earlier versions of the proposed text. Nonetheless, there are provisions within the text around the significance of global equity. These could be interpreted in ways that facilitate global responses, but much will depend on how these are implemented in practice.

Building upon this, Part III makes the case that such individualized approaches to the use of IPRs are an institutionalized feature of the current IP system where key actors, including rightsholders and states (or regions), have institutional goals that often align towards (and embed) such priorities. Such issues are compounded by the siloed nature of relevant legal systems related to IPRs and other related areas. Accordingly, for effective change to be achieved around how IPRs over health technologies are conceptualized and used during pandemics, including in the implementation of relevant provisions on IPRs in the WHO Pandemic Agreement (May 2025) or other relevant initiatives in future pandemics, Part IV argues that bottom-up approaches are needed. It argues that a key element to this is around reconceptualizing how resources needed for the development of health technologies are recognized in the health innovation system. It will argue that access to such resources (such as public funding, biobank samples, etc.) needs to be leveraged by relevant intermediary bodies (so-called resource providers) via the adoption of conditions around access to their resources, which mandate pathways towards equitable access to health technologies developed particularly in pandemics. However, ideally such actions would need to be targeted in a collective manner i.e. taken by a majority of relevant actors across relevant states, otherwise one could have forum-shopping to access resource providers who do not take such actions. Part V concludes by reflecting on the importance of embedding legal approaches to IPRs over health technologies needed for pandemic preparedness and response to align with the prioritization of global health needs around access to health technologies. Such responses should recognize IP rightsholders’ interests, but do so in a way that balances and also recognizes the role of other resource providers, and the importance of global access to such technologies in tackling pandemic threats.

## Part I: No [One] Is an Island: The Folly of Individualized IPRs Approaches Around Access to Health Technologies During Pandemics — A Focus on COVID -19

There have been extensive discussions around the role and operation of IPRs over elements of COVID-19 health technologies, including diagnostics, medicines, and vaccines, during the pandemic.^18^ This article does not seek to replicate such discussions. Instead, it provides a brief overview of the role of IPRs in health contexts, and ways that IPRs, focusing on patents, impacted the distribution and allocation of COVID-19 health technologies. This overview is offered solely for the purposes of providing necessary background for the arguments that follow.^19^ In doing so, section (i) will briefly highlight how IPRs can potentially be used in ways that impact the development, delivery and access to health technologies, including but not exclusively in pandemic contexts. Following this, section (ii), reflecting on the COVID-19 context, will argue that rightsholders and states (or regions such as the EU) in many cases adopted an individualized approach to the use of IPRs over health technologies and agreements related to access to vaccines during the COVID-19 pandemic, prioritizing their own or national/regional interests first. It highlights the significant impacts of this for both LMICs and HICs, including considering scientific evidence, which shows how such nationalistic/individualistic approaches led to excess deaths and increased the likelihood of new virus strains, which could have threatened pandemic response more generally globally.

### (i) IPRs and Access to Health: Tensions and Debates

IPRs, including patents, can act as a double-edged sword in the health innovation context:^20^ On the one hand, the exclusive rights offered by patents (and other IPRs) can incentivize the development of health technologies. ^21^ For example, a patent must be granted for a minimum of twenty years in World Trade Organization states.^22^ Where granted, a patent enables the rightsholders to exclude others from various aspects of using the patented technology for commercial purposes (such as making, supplying, selling that technology, etc.) without the rightsholders’ permission. Accordingly, patents provide rightsholders the ability to derive an income stream from a technology. For instance, patents can be licensed for use by third parties for monetary or other return. Moreover, patents (and other IPRs) are increasingly discussed as assets or collateral in themselves,^23^ used to attract investment and loans.^24^ Furthermore, various types of IPRs — such as patents, copyrights, trade secrets, etc. — are often used over different elements of technologies, including health technologies, in an overlapping manner to maximize the IP protection rightsholders have.^25^

However, alongside this incentivization role of IPRs, the exclusive nature of IPRs enables rightsholders to hold a governance function over patented technologies.^26^ Rightsholders can control key aspects of who has permission to use their patented technology, including to make the patented medicine, and who can use such patented technologies (for commercial purposes) in other products (including, where needed, for other health technologies). Accordingly, rightsholders can use patents (and other IPRs) in a way that enables them to dictate what actors (including states) can access a patented technology first, including in cases of limited supply, such as during health emergencies.^27^ Using a patented technology without the rightsholders’ permission, aside from some exceptions,^28^ could lead to patent infringement litigation, which can act as a deterrent for unauthorized production/replication of patented technologies. Patents (and IPRs) can enable rightsholders to hold a monopoly role over the technology. For example, by refusing licenses to other parties, rightsholders may become the sole provider of a technology, which can limit supplies and enable them to exert higher prices. This can impact access to health technologies, particularly in cases of global demand for health technologies such as during pandemics.^29^ In such cases, in many instances under the current system, as will be discussed below, LMICs with limited economic powers/resources are likely to fall behind HICs in gaining access to such health technologies.

Such issues have been exacerbated in the thirty years since the commencement of the TRIPS Agreement in 1995 (adopted in 1994 and commenced on January 1, 1995).^30^ Amongst other provisions, article 27 of TRIPS provides that patents must be made available in all fields of technology, which includes the health field. Prior to the TRIPS Agreement, WTO states could tailor national systems including, for example, by abolishing patents over certain fields where needed for national economic or health needs. However, post-TRIPS this is not possible for TRIPS Contracting States — and as TRIPS is required for participation in the WTO system, all WTO states must abide by the TRIPS Agreement. The TRIPS Agreement contains several “flexibilities,” including provision for states to adopt compulsory licenses (CL) where needed in a range of contexts. States’ ability to use such “flexibilities,” including in public health contexts, was confirmed by the Doha Agreement (2001).^31^ Nonetheless, significant limitations remain around the balance between IPRs and health in terms of such flexibilities and how they operate in practice in such contexts.^32^ A detailed examination of such limitations is beyond the scope of this article; however, reflecting further on the COVID-19 context illustrates some of the challenges relating to IPRs and public health, particularly in health emergency contexts.

### (ii) COVID-19: IPRs & Individualized Approaches to the Distribution of COVID-19 Health Technologies

During the COVID-19 pandemic, a significant gap emerged between HICs and LMICs around access to COVID-19 health technologies, including vaccines. A key factor contributing to this was how IPRs operated over elements of such technologies. If we consider, for example, COVID-19 vaccines, from the early stages of COVID-19 being declared a pandemic (March 2020) by the World Health Organization (WHO), there was a global race to develop vaccines that would be safe and effective against COVID-19.^33^ During that early phase of the pandemic, concerns grew around to what extent a vaccine — if developed — would be available in all countries globally, particularly for LMICs. In 2020, issues arose around access to diagnostics and therapeutics against COVID-19, with several instances whereby once a therapeutic was seen as having potential against COVID-19, the majority of supplies of that therapeutic were often purchased by a HIC(s), with limited supplies remaining for other countries.^34^

Against this backdrop, on October 2, 2020, with increasing concerns that LMICs would be left behind in terms of access to vaccines and other health technologies, a group of LMICs led by India and South Africa put forward a proposal to waive IPRs over COVID-19 health technologies.^35^ This proposal stated that:

There are several reports about intellectual property rights hindering or potentially hindering timely provisioning of affordable medical products to the patients…. In addition, many countries especially developing countries may face institutional and legal difficulties when using flexibilities available in the Agreement on Trade-Related Aspects of Intellectual Property Rights (TRIPS Agreement)…

This original waiver proposal was supported by a significant number of LMICs and other groups; however, ultimately, it met with limited success during the pandemic.^36^ It was met with significant opposition from HICs. Eventually a much-watered-down version of a waiver was agreed on June 2, 2022 (hereafter referred to as the QUAD outcome text).^37^ However, the adopted QUAD outcome text shows limited resemblance to the original waiver proposal. For instance, it did not apply to all IPRs. It only applied to patents, it also only applied to COVID-19 vaccines, and was only applicable in limited circumstances.^38^ Moreover, the QUAD outcome text was adopted nearly two years after the original waiver proposal was published, despite the fact that this original proposal was put forward during a global pandemic. Other voluntary initiatives to encourage the sharing of IPRs and technology transfer over COVID-19 vaccines (and other health technologies) also met with limited, if any, success during the pandemic.^39^ For example, the WHO COVID-19 Technology Access Pool (C-TAP), which was established in the spirit of solidarity, aimed to set up mechanisms for the voluntary sharing of IPRs and facilitation of technology transfer to enable a rapid scale-up of COVID-19 technologies given the health emergency. Yet C-TAP had limited success during the pandemic, and a key obstacle was limited interest/support from rightsholders in participating in C-TAP, including in the COVID-19 vaccine context,^40^ and lack of support from HICs.^41^

Indeed, even prior to when the first COVID-19 vaccines were approved for use (the first vaccine was administered in January 2021 in the UK),^42^ HICs had already negotiated advance purchase agreements with the relevant rightsholders to obtain several doses of approved vaccine(s) for each member of their national population.^43^ As vaccines started to become available in HICs, many LMICs waited for supplies via the COVAX system, which experienced significant difficulties in obtaining vaccine supplies during the early stages of the pandemic.^44^ Effectively, commitments for multiple doses of vaccines per population were obtained by HICs in many cases, whilst LMICs had to wait for any supplies to provide first doses to their populations, including healthcare workers in LMICs.^45^ The resulting vaccine inequity between HICs and LMICs was criticized widely during the pandemic, including by the WHO.^46^ In January 2021, Dr Tedros Ghebreyesus, WHO Director General, stated that the inequity emerging put the world on the brink of a “catastrophic moral failure.”^47^

IPRs were not the only factor that impacted the distribution and scaling up of COVID-19 vaccines during the pandemic.^48^ However, IPRs were a key element of this. Moreover, IPRs were a key factor that enabled HICs to secure greater doses than LMICs. Such IPRs enabled rightsholders to act as key private governance actors allowing them to control key elements of the distribution of patented technologies (or other IPRs), whilst contracts were then used by rightsholders to specify the terms of such distribution.^49^ Moreover, vaccines and other health technologies are typically protected by multiple patents and other IPRs.^50^ Hence, permissions from multiple rightsholders, and relevant know-how, may be needed in such contexts to produce such vaccines.

Furthermore, the existence of TRIPS flexibilities, including compulsory licenses (CLs), had limited effects in alleviating such IPR issues during the pandemic.^51^ As discussed extensively elsewhere, whilst some states modified national laws to facilitate use of such flexibilities and other emergency powers,^52^ in practice, CLs have significant limitations to address access to vaccines issues during pandemic contexts.^53^ These include the fact that many CLs operate in contexts where national requirements for the issuance of the CL may be bureaucratic and time-consuming, states may fear backlash or sanctions related to the use of CLs, and these must typically be applied for individually in each state. Relatedly, CLs only apply to patents and do not apply to other IPRs that will be applicable over emerging health technologies.^54^ Furthermore, although the Doha Declaration (2001) reiterated states’ ability to use CLs or other flexibilities in TRIPS to address health needs, including, but not limited to, health emergency contexts,^55^ difficulties remain in using such flexibilities.

The inequity which arose around lack of supplies of COVID-19 vaccines and other health technologies which LMICs could obtain during the pandemic had significant effects. For example, it is estimated that the vaccine inequity likely caused many excess deaths during COVID-19.^56^ Moore et al. (2022) used global modelling to examine the likely impact of the global inequity and attempts to address this during the pandemic, and they argued that:^57^

We calculated that increased vaccine sharing, without any changes to NPIs [non-pharmaceutical interventions], would have substantially reduced COVID-19 infection mortality in lower-income countries, although some high-income countries would have had increased mortality unless additional measures were taken. Overall, we estimate that this vaccine sharing scenario would have prevented 1.3 million deaths worldwide (as a direct result of COVID-19) by the end of 2021, although this figure could be substantially increased if increased vaccine sharing from high-income countries had been compensated for with slower easing of NPIs.^58^

Indeed, evidence suggests that not only did global vaccine inequity likely lead to many excess deaths globally, the approach, which prioritized national vaccine allocation over global distribution, was also potentially contrary to ideal public health strategies around tackling the pandemic at a global level. This is because scientific evidence suggests that leaving areas without vaccine coverage — or with limited vaccine coverage, as happened in many LMICs during COVID-19 — has the potential to increase the risks of a new strain of COVID-19 emerging; such new strains could have been resistant to existing vaccines, and hence, this could have placed the global response to COVID-19 at risk.^59^ In 2022, based on research using a multi-strain metapopulation model and COVID-19, Ye et al. argued that:

… vaccine inequity provides only limited and short-term benefits to HICs, whereas it leads to moderate increases in infections and deaths in LMICs. However, such increases may result in elevated risk of future waves (caused by new strains) affecting not only LMICs but also HICs. A sharper disparity in vaccine allocation between HICs and LMICs leads to earlier and larger peaks in pandemic size in future waves.^60^

Similarly, Moore et al. stated in their 2022 article that:

… preventing the rapid emergence of new variants and, hence, the long-term control of COVID-19 relies on reducing the global burden of infection, creating a tension between national (short-term) and international (long-term) perspectives that is greater now than at any time in the pandemic.^61^

Thus, our recent experiences from the COVID-19 pandemic illustrate the need for rapid global responses to the scaling up of vaccines (and other pandemic health technologies) and, critically, the need for such vaccines to be distributed based on global health needs. Looking to future pandemics, based on their findings, Moore et al. (2022) argued that: “The message for any emerging outbreak is clear: distributing vaccines across the globe proportional to need, rather than to wealth, will have beneficial effects for all.”^62^

At this juncture, it is important to turn to considering future pandemic preparedness measures and the extent to which such lessons have been embedded in these measures in the context of provisions relating to IPRs and access to pandemic technologies within the recently adopted WHO Pandemic Agreement.

## Part II: Pandemic Preparedness Negotiations Around IPRs in the WHO Pandemic Agreement: The Zero Draft Text vs. the Agreed April 2025 Pandemic Text

Against the ongoing backdrop of the COVID-19 pandemic, world leaders announced the intention to establish a pandemic preparedness and response instrument in March 2021.^63^ In October 2021, a zero-draft report was published by the “Working Group on Strengthening WHO Preparedness for and Response to Health Emergencies.”^64^ The Report focused on:

… the assessment of the benefit of a new WHO convention, agreement and other international instrument on pandemic preparedness and response to be submitted to the special session of the World Health Assembly (WHASS) on 29 November–1 December 2021.^65^

This zero draft report highlighted that several key areas for pandemic preparedness and response fell outside the scope of the International Health Regulations (2005) and “…may be best addressed either through a potential new instrument or through another normative, policy or programmatic tool available through WHO.”^66^ It stated that Member States had raised several issues, including equity around access to health technologies during pandemics and related issues, including IPRs. More specifically, the report stated that Member States raised:

(b) Equity, including … *intellectual property, technology transfer and empowering regional manufacturing capacity* during emergencies to discover, develop and deliver effective tools and technologies. While each of these areas are complex, equity is at the core of the breakdown in the current system and *is ideally suited for negotiation under the umbrella of a potential new instrument.* [Emphasis added]

This statement showed a clear recognition of the need to consider IPRs as part of a discussion on equity in pandemic preparedness actions. It suggested a desire by (at least some) WHO Member States to address potential issues around IPRs and equity in this context.

In December 2021, the World Health Assembly agreed to commence the process towards developing such an instrument.^67^ It established an Intergovernmental Negotiating Body (INB) established by WHO Member States with the aim that this INB would draft and negotiate a pandemic preparedness instrument under the WHO framework (article 19 of the WHO Constitution provides for its ability to adopt such instruments/initiatives).^68^ The negotiation of that instrument involved thirteen rounds of meetings, with several revised versions of a text emerging as part of these. These negotiations culminated on April 16, 2025, in an agreement on the adoption of a text. Following this, WHO Member States formally decided by consensus to adopt the Pandemic Agreement text at the 78th World Health Assembly meeting on May 20, 2025, through resolution WHA78.1.^69^ Nonetheless, even though this text was adopted in May, certain aspects still require discussion and finalization, including the need to obtain agreement amongst states on the details of a proposed pathogen access and benefit sharing (PABS) agreement, which has been included in the annex that needs to be agreed by states in future.^70^

Notably, although this Pandemic Agreement has been agreed by WHO states, the US did not participate in this process, and this could likely reduce the effect of this agreement, particularly given the role of the US within the health innovation landscape and that many pharmaceutical companies are based in the US.^71^

Nonetheless, the fact that it was possible to reach any consensus on the Pandemic Agreement, and that this was achieved in three years, which is a relatively quick time frame in terms of such international agreements, could be seen as an achievement.^72^ Having said this, this section will argue that the specific provisions agreed upon in the context of IPRs are considerably watered down from the original ambitions. Whilst there are a range of references to equity, and provisions within the text that could be used to deliver pathways towards better global access to health technologies in future pandemics, the wording of these provisions leaves considerable discretion to states and the international community. Thus, much will depend on how these provisions are implemented in practice.

In the section that follows, the analysis focuses on the published first version of the Pandemic Agreement text (hereafter “Zero Draft text”) by the INB and the recently adopted text, the WHO Pandemic Agreement (May 2025). Several other versions of the text were negotiated between these stages; however, for the purposes of space, these are not considered here. Moreover, this analysis focuses primarily on the provisions related to IPRs for future pandemics and not on broader aspects.^73^

The Zero Draft text of the Pandemic Agreement was published on February 1, 2023, for consideration as part of the fourth meeting of the INB.^74^ This zero-draft instrument contained ten references to the term “intellectual property,” two references to patents, and ten references to “equity.” In contrast, the adopted Pandemic Agreement (May 2025) contains only five references to intellectual property, one reference to patent(s) and six references to “equity.”^75^ Moreover, an analysis of the specific references to IPRs in both instruments demonstrates differences between the ambition of the original text in the Zero Draft text, and what could be agreed upon by Member States in the Pandemic Agreement (May 2025).

### (i) Preamble, Principles & Aims

First, it is useful to consider the spirit and aims of the agreements by comparing the preamble of the Zero Draft treaty text with the WHO Pandemic Agreement (May 2025) text. There were several references to the benefits and the potential impacts of IPRs on access to health technology within the first Zero Draft text, including under recitals 40-42, which recognized various elements, including the potential negative impacts of IPRs on the prices of health technologies, alongside recital 43, which stated that:

Recognizing the concerns that intellectual property on life-saving medical technologies continues to pose threats and barriers to the full realization of the right to health and to scientific progress for all, particularly the effect on prices, which limits access options and impedes independent local production and supplies, as well as noting structural flaws in the institutional and operational arrangements in the global response to the COVID-19 pandemic, and the need to establish a future pandemic prevention, preparedness and response mechanism that is not based on a charity model …

In contrast, the WHO Pandemic Agreement (May 2025) has limited references to the potential adverse implications of IPRs on access to health technologies, having removed the broader statements around this contained in the earlier drafts. Recital 16 states:

Recognizing that intellectual property protection is important for the development of new medicines *and recognizing the concerns about its effects on prices*, and recalling that the World Trade Organization Agreement on Trade-Related Aspects of Intellectual Property Rights (TRIPS Agreement), does not and should not, prevent Member States from taking measures to protect public health, and which provides flexibility to protect public health, as recognized in the Doha Declaration on the TRIPS Agreement and Public Health. [Emphasis added]

This contrast between the preamble texts of both versions could be seen as suggesting a change of focus and ambition around IPRs. Furthermore, whilst changes in international treaty language are to be expected between the first draft and adopted final text, given the context of COVID-19 and the need for collective action it highlighted, it raises uncertainty around how potential obstacles posed by IPRs and access to health technologies will be addressed in future pandemic preparedness initiatives.

Alongside the preamble to the text, in terms of key principles within the adopted WHO Pandemic Agreement (May 2025) text, there are several references to the importance of “equity” in pandemic preparedness. For example, article 3(4) states that:

3. To achieve the objective of the WHO Pandemic Agreement and to implement its provisions, the Parties shall be guided, inter alia, by the following:

… 4) equity as a goal, principle and outcome of pandemic prevention, preparedness and response, striving in this context for the absence of unfair, avoidable or remediable differences among and between individuals, communities and countries …

This provision suggests a commitment to equity within the Agreement; however, it is not clear how “equity” will be defined or implemented in this context, and only time will tell how effective this provision will be. Moreover, whilst a full examination of the proposed Pathogen Access and Benefit Sharing System (PABS) set out in the Pandemic Agreement (May 2025) is beyond the scope of this article, in terms of global distribution under article 12, there is a requirement that participating manufacturers make available to the WHO 20% of their vaccines, therapeutics and diagnostics, with 10% needed to be made available to the WHO as a donation and the remainder reserved at affordable prices to the WHO. Including a required minimum amount for donation is welcome; however, 20% is a relatively limited amount, with only 10% offered as a donation in such contexts.

### (ii) Provisions on Access to Technology: IPRs and Technology Transfer

Turning then to specific provisions related to IPRs and technology transfer, article 7 of the Zero Draft text was entitled “Access to technology: promoting sustainable and equitably distributed production and transfer of technology and know-how.” Article 7(4)(a) stated that:

4. In the event of a pandemic, the Parties: (a) will take appropriate measures to support *time-bound waivers* of intellectual property rights that can accelerate or scale up manufacturing of pandemic-related products during a pandemic, to the extent necessary to increase the availability and adequacy of affordable pandemic-related products …

There is no reference to a waiver of IPRs in the adopted WHO Pandemic Agreement (May 2025) text. Having said this, article 11(3) of the adopted text states that:

The Parties shall cooperate, as appropriate, *with regard to time-bound measures* to which they have agreed within the framework of relevant international and regional organizations to which they are a party, to accelerate or scale up the manufacturing of pandemic-related health products, to the extent necessary to increase the availability, accessibility and affordability of pandemic-related health products during pandemic emergencies. [Emphasis added]

The reference to time-bound measures under article 11(3) provides an explicit acknowledgment that states can decide on temporary measures, which could potentially include waivers of IPRs if “necessary” to increase access, etc., to health technologies. However, the lack of reference to waivers or to IPRs in this context means states would have to exercise their discretion to adopt these. Moreover, the reference to waiver of IPRs under article 7(4) of the Zero Draft, although not without limitations,^76^ was framed more akin to an obligation on states, who in the event of a pandemic “will take appropriate measures to support *time-bound waivers* of intellectual property rights…” [Emphasis added]. In contrast, article 11(3) of the adopted WHO Pandemic Agreement (May 2025) text states “The Parties *shall cooperate*, *as appropriate*, with regard to time-bound measures…” [Emphasis added]. The use of the term “shall cooperate” together with the term “as appropriate” suggests considerable leeway for states in this context. Much may depend on whether and to what extent such actions are seen as “appropriate” and in what contexts, and what is considered “cooperation” in this context.

Article 7(4)(c) of the Zero Draft text stated that:

In the event of a pandemic, the Parties: (c) shall encourage all holders of patents related to the production of pandemic-related products *to waive, or manage as appropriate, payment of royalties* by developing country manufacturers on the use, during the pandemic, of their technology for production of pandemic-related products, and shall require, as appropriate, those that have received public financing for the development of pandemic-related products to do so … [Emphasis added]

There is no explicit reference to a waiver of royalties in the WHO Pandemic Agreement (May 2025 text). Article 11(1)(d), however, provides that states should encourage rightsholders (of relevant patents or licenses for pandemic-related health products) to “forgo or otherwise charge reasonable royalties in particular to developing country manufacturers during a pandemic emergency,…” Nonetheless, this wording limits the effect of this provision, as it presents an option to either forgo or charge reasonable royalties. It is not clear how “reasonable” royalties would be determined in such contexts, and even if a royalty appears reasonable for a HIC this may be more than what could be afforded by LMICs. If the current IPR model applies, there is also likely to be limited incentives for rightsholders to offer such technologies to LMICs at lower prices than they may be able to obtain from HICs in cases of global shortages.

In this context, the general text of article 11(1) of the WHO Pandemic Agreement (May 2025) on technology transfer and “cooperation on related know-how for the production of pandemic-related health products” is useful to consider in full. It states that:

Art 11(1): Each Party shall, in order to enable the sustainable and geographically diversified production of pandemic-related health products for the attainment of the objective of the WHO Pandemic Agreement, as appropriate:

- *promote* and otherwise facilitate or incentivize, transfer of technology *as mutually agreed*, including transfer of relevant knowledge, skills, technical expertise, and cooperation on any other related know-how for production of pandemic-related health products, in particular for the benefit of developing countries, through measures which may include, inter alia, licensing, capacity building, relationship facilitating, incentives or conditions linked to research and development, procurement or other funding and regulatory policy measures;
- take measures to *enhance the availability of licenses* for pandemic-related health technologies *to which it owns the rights*, on a non-exclusive, transparent and broad geographic basis and for the benefit of developing countries, where and as feasible, in accordance with national and/or domestic law, and international law and encourage private rights holders to do the same;
- take measures *to publish, in a timely manner the terms of its licensing agreements* relevant to promoting timely and equitable global access to pandemic-related health technologies, in accordance with applicable law and policies, and shall encourage private rights holders to do the same;
- *encourage holders of relevant patents or licenses for the production of pandemic-related health products to forgo or otherwise charge reasonable royalties* in particular to developing country manufacturers during a pandemic emergency, with the aim to increase the availability and affordability of such products to populations in need, in particular people in vulnerable situations;
- *promote* the transfer of relevant technology as mutually agreed, including transfer of relevant knowledge, skills and technical expertise, for pandemic-related health products by private rights holders, to established regional or global technology transfer hubs, coordinated by WHO, or other mechanisms or networks; and
- during pandemic emergencies, encourage manufacturers to share information, relevant to the production of pandemic-related health products, in accordance with national and/or domestic law and policies. … [emphasis added]

The obligations under article 11(1)(a), (d), and (e) are framed as obligations on states to “promote” and “encourage,” which may have limited teeth in practice. Moreover, the language used in article 11(1)(b) is more onerous as it provides that states would “*take measures to enhance the availability of licenses* for pandemic related health technologies *to which it owns the rights.*” However, it is not clear what is meant by “measures” in this context, and this provision appears to refer only to IPRs over technologies which states hold relevant rights, not to other rightsholders, although it does suggest that states also encourage private rightsholders to do the same. It also leaves states with considerable discretion as it is not clear what “enhancing” availability would entail. Furthermore, article 11(1)(c) is welcome as a measure to encourage transparency around licensing agreements and the terms within these, as is the reference to “encouraging” private rightsholders to do the same. However, states may have limited powers to enforce such mechanisms against such rightsholders. More generally on this point, it is not clear how, if at all, these provisions would be monitored, and whether there are any consequences for failure to abide by such measures.^77^

Reflecting on the IP/technology transfer provisions within the Pandemic Agreement (May 2025), it is important to bear in mind that there was significant debate over these provisions, and at times the opposition to the original provisions around technology transfer, particularly from HICs and regions, looked like this could lead to the collapse of the negotiations.^78^ Arguably, the issues related to IPRs/technology transfer, and the fact that the final text is more limited than earlier versions, is another example of prioritization of national interests and priorities by states, despite the implications of this that was seen during the COVID-19 context.

Nonetheless, there are positives, which should be acknowledged in relation to provisions related to IPRs in the Agreement. For example, ‘t Hoen, previously commenting on the final April text of the Agreement (which is identical to the adopted May text), highlighted the definition of “as mutually agreed” under the Agreement, which is applicable for article 11 and is particularly important. The April text and the adopted WHO Pandemic Agreement (May 2025) define this as follows: “‘as mutually agreed’ means willingly undertaken and on mutually agreed terms, without prejudice to the rights and obligations of the Parties under other international agreements.” t’ Hoen has argued that this definition recognizes “that if the willingness to ‘mutually agree’ is not there, governments can take other measures to make technology transfer happen.”^79^ This definition is welcome, as it leaves the door open to states to adopt mandatory measures if needed. Nonetheless, much will depend on how this provision is interpreted in practice. Critically, in the event of a future pandemic, much will also depend on how willing states are to use alternative mechanisms if voluntary agreements cannot be reached. Based on past practices, it is likely that states that lack access to technologies will be LMICs, and there are a range of obstacles that such LMICs have faced in taking alternative action in the past, especially where international action is sought and needs support of other WHO states including HICs. Moreover, whilst the WHO Pandemic Agreement (May 2025) text does not stop states/the international community adopting other mechanisms, on a more pessimistic view, arguably it could be seen as adding little in terms of mandating states to adopt clear steps to tackle IPR issues, which can arise in relation to health technologies in pandemic contexts.

### (iii) Reflecting on the WHO Pandemic Agreement (May 2025): The Importance of Implementation

Reflecting on the final WHO Pandemic Agreement (April 2025) text (which was the version ultimately agreed by WHO Members and became the adopted WHO Pandemic Agreement (May 2025)), ‘t Hoen has argued that:

Compared to the ambitions and expectations of three years ago, the outcome is not as strong as it should be. While all key issues are addressed in one way or another, there are few hard new obligations for member states. Many of the provisions are couched in non-committal language, allowing for action only “when deemed appropriate,” for example. Such terms leave it unclear what the actual obligation of member states is.^80^

In short, many of the provisions within the agreed WHO Pandemic Agreement text related to IPRs focus on voluntary non-binding requirements on states, with considerable discretion left to states in relation to equitable access to pandemic health technologies. Whilst we can hope that systems for delivering access to pandemic health technologies would develop more effectively if we face a future pandemic than happened in COVID-19, nonetheless, our recent experience of COVID-19 raises considerable uncertainties. Indeed, the May 2025 Lancet editorial discussing the agreed WHO Pandemic Agreement text argued that:

… the lack of accountability, coupled with the weak requirements on health technologies, means that the treaty will be unable to prevent repetition of one of the key failures seen during COVID-19 — the voracious acquisition of key resources by a handful of powerful actors at the expense of all. When the next pandemic does arise, it will take more than the pandemic treaty to ensure a truly equitable response.^81^

Reflecting on the WHO Pandemic Agreement and COVID-19 more generally, three broader questions arise: (1) What were the key legal drivers contributing towards nationalistic approaches to vaccine allocation during the COVID-19 pandemic?; (2) Relatedly, why, given the recent experiences in the COVID-19 context including the inequities that arose around IPRs and pandemic health technologies, and the risks this poses for the pandemic response more generally due to this, have we seen such nationally divided responses to the IP/technology transfer provisions in this WHO Pandemic Agreement process?; (3) And, looking to the future, building upon this recently adopted WHO Pandemic Agreement, what types of strategies can be used to seek to encourage an institutional change that would strengthen the likelihood of this Agreement being implemented in a way that recognizes global health needs in pandemic contexts, and which ideally offers additional pathways to support global equitable access to vaccines (and other health technologies). The article will now turn to consider such issues.

## Part III: “Entire of Itself”: An Institutionally Embedded Individualized Approach to IPRs and Procurement of Health Technologies During COVID-19

This section will consider the potential legal drivers towards nationalistic responses to vaccine allocation during the COVID-19 pandemic, and how, despite COVID-19 experiences, nationalistic approaches are arguably still apparent in discussions on future pandemic preparedness around technology transfer and IPRs in the WHO Pandemic Agreement. It will argue that such responses are at least in part symptomatic of an institutional system that has embedded legal and political factors that tend towards prioritizing individual (national states, regions, and/or rightsholders) needs, and which are not aligned towards longer-term collective global health needs.

Three key elements to this argument are proposed: (1) It highlights that IP rightsholders who have a key role in deciding who can access patented technologies first and on what terms in many cases are for-profit companies or use for-profit companies to license IPRs. This will arguably impact how rightsholders use IPRs during pandemics, as where such discretion of rightsholders is left unfettered, it will likely be used in ways that align with distribution of vaccines based on profit maximization strategies. Thus, it is likely states with greater purchasing powers will gain access first to IP-protected technologies, without external drivers countering this. (2) It argues this is compounded as states and regions are also typically driven to prioritize national/regional needs first, due to legal, political, and other broader institutional drivers. Such drivers will likely often tend towards the prioritization of national (short-term) needs rather than global long-term needs, and this may be compounded in cases of uncertainty such as pandemic contexts. (3) It argues that this approach is further embedded by the insular nature of international IP law, which in many ways operates at an international level in a silo i.e. away or separate from other legal fields such as human rights, which engage with the broader health implications of certain uses of IPRs. At the international level, arguably human rights approaches have had limited practical effect to date in changing the international community’s priorities toward global equity in access to pandemic health technologies. This point is not suggesting human rights approaches can never have success in facilitating global equitable access to pandemic health technologies; rather, it is arguing that in the current institutional context and within the current health innovation landscape, to date such approaches have had limited success.

### (i) Rightsholders – Legal Drivers Towards Profit Maximization

First, as discussed elsewhere, rightsholders of IPRs, including in the health technology context, in many cases are private companies that typically operate towards goals of profit maximization.^82^ For instance, in common law countries, companies operate based on the shareholder primacy model, typically understood as requiring them to maximize shareholder “value,” often interpreted as profits for shareholders.^83^ Moreover, even where IPRs are held by publicly funded universities, increasingly, universities use IPRs to generate revenue and income. In some countries there has been a trend towards the use of surrogate licensing companies, which license university IPRs on a profit basis, or in some cases the practice is to commercialize university IPRs through spin-out companies created for such purposes.^84^ Furthermore, under many employment contracts, including contracts for university employees, where scientific research may be developed, IPRs generated in the course of the employment are often assigned to the employer, such as the university or company. In such cases, it is the employer that will decide on how to use such IPRs. Accordingly, in many instances it is companies that will hold or license IPRs for health innovations, including for pandemic health technologies.

Where companies are driven to use company assets, which includes IPRs, in ways that maximize profits, it is unsurprising that during the COVID-19 pandemic entities with greater resources were able to obtain access to pandemic health technologies first. A limited number of rightsholder companies agreed to share IPRs within the C-TAP to facilitate the rollout of vaccines during the pandemic. Indeed, without an external driver such as a law mandating the sharing of IPRs in such context, to offer to voluntarily share IPRs could be contrary to companies’ aims around profit maximization. Moreover, whilst companies have social responsibility obligations, in many cases these are non-binding, and uncertainty can arise around what such obligations entail, including the extent to which these impact IPRs usage by companies, particularly given the complexity of issues at stake in such contexts.^85^

### (ii) National States and Regions: Drivers Towards Maintaining the Status Quo of Prioritizing National Short-Term Interests

Second, national/regional responses to securing access to pandemic health technologies have also demonstrated an (understandable) focus on prioritizing their populations’ needs in pandemic or other health-related emergency contexts. Where there is a shortage of vaccines available to administer globally against a pandemic, the general populations within states would likely expect their government to seek rapid access to and provision of vaccines and other treatments for their populations. Citizens may support the provision of vaccines and other pandemic technologies to other states.^86^ Nonetheless, without external drivers to encourage or mandate vaccine rollout that includes a prioritization of global health needs, governments may be reluctant to take actions that could be seen as prioritizing collective global needs over national needs in obtaining vaccines (and other health technologies), especially in early stages of a pandemic where considerable uncertainties remain about a disease. Furthermore, states have relevant human rights obligations to their own populations including obligations under the right to health, and part of this obligation is around delivering accessible health technologies, including vaccines.^87^

In pandemic times, a strong case can be made that prioritizing the global distribution of vaccines (as opposed to a vaccine rollout prioritizing national interests based on those with the highest purchasing powers) aligns with both national and global interests. As discussed above, prioritizing global vaccine allocation during pandemic context could reduce risks of spread of the pandemic and reduce the risks of new variants. However, such responses driven by prioritizing global needs may result in fewer vaccines going to the national states in some HICs in the short term (as vaccines supplies are allocated across more states) and, as discussed above, depending on the context, it may potentially give rise in the short term to an increase of infections/deaths at the national level. Furthermore, such situations could lead to uncertainty for states around what prioritizing vaccines for global health needs or towards a more globally distributed roll-out (instead of national needs first) may mean in the short term for their national populations. Concerns may include uncertainties around the mortality rate of any new pandemic, and whether potential delays in obtaining vaccines for national states entailed by prioritizing global pathways could lead to higher risks for national populations. States may also be concerned about whether other states will cooperate — a concern may arise around whether, even if they adopt such strategies, other states will also fully commit to such strategies or some states will seek alternative additional pathways to gain priority access.^88^

In such contexts, institutional theories suggest that where institutions (which can be broadly defined, and could include, for example, government or departments of that government responsible for certain aspects) have discretion on how to operate,^89^ and particularly in times of uncertainty,^90^ then they are typically institutionally driven to act in ways which prioritize their core aims. For example, MacCormick has argued that institutions typically function in line with the core aim/end of that institution.^91^ He noted that “an explanation of any institution requires an account of the relevant rules set out in light of its point.”^92^ Pandemics and questions around allocation of vaccines within emergency contexts or for pandemic preparedness instruments gives rise to considerable discretion for states (under the current model) in how to act to address these, and pandemics also by their nature give rise to considerable uncertainty, risks, and fear of populations within states. Thus, institutional theories might suggest states in such contexts will focus on prioritizing their core aims, which are around protecting national interests, including in access to vaccines.

Whilst this explanation of institutions — and hence institutional decision-making patterns — does not mean that such institutions cannot adapt to operate under different contexts or purposes to the stated core aim, nonetheless, in such cases, change may be difficult to achieve, as “it is the institution that normally functions towards a given broadly-stated end — its ‘final cause’ — that is so adapted.”^93^ One might argue that if the certainty increases around the need to ensure global roll-out to address the pandemic, this could lead to change; however, it is questionable how much evidence states would need of this, or in what circumstances, to adopt such longer-term views.

Reflecting on such drivers and aims of national governments at least in part explains moves towards vaccine nationalism during COVID-19, even in cases where collective global strategies based on health needs were suggested by scientific evidence. Moreover, such institutional factors may explain at least in part why HICs that may be able to access pandemic technologies under the current health innovation model may oppose provisions around IPRs that mandate IP sharing/waiving etc. where needed during pandemics. It also may explain why such provisions are often supported by some LMICs, which in many cases are unable to obtain access to such technologies until much later than HICs under the current system. Arguably, these issues are reflective of a much deeper fundamental tension between the needs and interests of HICs and LMICs that has been embedded within the current TRIPS system, and within the broader health innovation landscape more generally. Such issues and tensions arguably also played out in the discussions on the WHO Pandemic Agreement. Thus, it is perhaps unsurprising that HICs may support the status quo, in many cases opposing provisions mandating technology transfer and sharing of IPRs during pandemics, and part of this may be around concerns that an alternative approach may pose uncertainties for such HICs. In contrast, LMICs that fell significantly behind HICs in the COVID-19 vaccine pandemic are arguably much more likely to be acutely aware this is legally possible and sadly, quite likely to happen again in future pandemics unless alternative approaches are developed.

Notably, in such discussions on national versus global needs in vaccine allocation including around the role of IPRs, a false dichotomy is arguably being created in certain contexts. This is because if states and rightsholders showed greater willingness to work collectively towards rapidly upscaling vaccine production, it is conceivable that a faster roll-out of vaccines is possible for all states, particularly when one considers what was achieved in terms of creating the vaccine for COVID-19. Yet strong political will, multilateral action and global solidarity across a range of public and private actors would be critical to the success of such an approach. All of these are difficult to achieve, particularly in the face of health emergencies when states face significant uncertainty and may revert to existing institutional modes of thinking, and this may lead towards a pattern of maintaining the status quo for states that can obtain access for their national population needs in such contexts.

Moreover, aside from questions around the likely impact of IPR sharing provisions or models on access to health technologies, under the current health innovation system, rightsholders are also likely to be opposed to such models, as these may impact the profits such rightsholders can obtain. Furthermore, such rightsholders are often large companies that in many cases are headquartered in HICs and are key economic entities that bring important financial and employment contributions to such HICs. This may be an additional factor, which could increase the likelihood of opposition of some HICs to provisions related to IP sharing or around the negative impacts of IPRs on pandemic health technologies.

### (iii) Mechanisms for Intervention at the International Law: Silos and Interpretative Gaps

Finally, such issues are likely compounded by the siloed nature of the international legal systems wherein the IPR system tends to operate within the trade context in a manner that is separate to other areas of law such as human rights.^94^ Furthermore, although debates over patents and access to health continue to give rise to questions around the relationship between IPRs and human rights,^95^ there are still considerable practical and institutional limitations in successfully using human rights at an international level to challenge approaches of states and rightsholders over access to IP-protected technologies during pandemic contexts.^96^ Indeed, Gostin et al. stated that:^97^

COVID-19 has created a catastrophic record of how human rights shortcomings undermine pandemic preparedness and response, and how health emergencies undermine human rights and fuel further violations.

A comprehensive examination of human rights issues and how these were addressed within the COVID-19 context is beyond the scope of this article. Instead, this section offers a brief reflection on some of the key challenges in using human rights to challenge vaccine nationalism and facilitate pathways towards global access during pandemic contexts. Four key (non-exhaustive) issues are highlighted here as factors under the current system which can limit the role of human rights at an international level to address vaccine nationalism during pandemic contexts.

First, TRIPS placed IPRs within the trade paradigm.^98^ In doing so, IPRs, including patents, are viewed under a trade lens, which tends to focus on the economic or trade-related elements of IPRs. Patents must be made available for all fields of technology, including health technologies. Yet, once granted, a patent on a health technology is viewed as fungible or interchangeable with patents over other technologies.^99^ This fungibility exists despite the impact that patents over health technologies can have given the nature of a health technology and that lack of access to such technologies can impact human health, and in some cases human life. The patent system under TRIPS arguably can become blinkered to the broader non-economic impacts of how patents can be used, and over time an institutional system has evolved in an insular nature which prioritizes the economic aspects of such rights.

Second, tensions existing between patents and access to health can give rise to potential questions around conflicts between rights, as patents (and other IPRs) are sometimes themselves viewed as part of a rights-based framework — for example, as part of a property right of the rightsholders — whilst access to health issues implicate the right to health and/or the right to life, etc. Yet it can be difficult to challenge uses of IPRs based on the potential impacts the use of such rights may have on third parties such as patients.

Relatedly, third, it can be difficult to prove that use of IPRs is contrary to the human right to health or life in practice, in certain contexts. Where raised, rightsholders may argue that patents (and other IPRs) are temporary in nature, that such IPRs provide incentives for the development of such technologies to recoup development costs and without which new technologies may not be available. There are also significant practical issues in pandemic contexts, including temporal issues with taking such challenges — human rights litigation can take time to develop, which is not conducive to the need for fast access to vaccines and other health technologies in pandemic contexts. Even where challenges are successful, the remedies that can be granted may be limited and may not necessarily address the access to health issues in the way applicants seek. Furthermore, litigation can be lengthy and costly.

Fourth, the human rights framework typically imposes binding obligations on national states, not on companies (who are the relevant rightsholders in many cases and whose actions may impact access to patented technologies). For example, under the right to health (which is protected under a range of international instruments, including article 12 of the International Covenant on Economic, Social and Cultural Rights (IESCR) which is the focus here) the obligation is on states to ensure obligations under the right to health are delivered. Moreover, states typically must abide by such obligations at a national level, and hence to people within that state rather than to those outside the state.

Other human rights obligations such as those relating to “nondiscrimination” and equality are relevant to global equitable access to health technologies.^100^ However, during the COVID-19 context, despite such rights having been acknowledged by various UN bodies and international groups as implicated in the vaccine inequity that arose,^101^ there were limited practical mechanisms achieved on foot of this that mandated obligations on states/international community to address such rights and deliver global access to vaccines and other health technologies. Moreover, states have obligations under international treaties, such as under the IESCR to take steps through “international assistance and cooperation” (IESCR article 2(1)) towards achieving economic, social and political rights, yet the lack of precision on what this entails for state obligations is problematic.^102^

These arguments are not intended to suggest human rights avenues or litigation can never be successful in pandemic or other access to health contexts,^103^ nor do they seek to discourage the role of human rights here. Rather, this section argues that challenging the (often) individualistic approaches to uses of IPRs over health technologies by states or rightsholders during pandemics via human rights law at the international level has limitations when done in the confines of the current institutional context.^104^

## Part IV: “Every[one] Is a Piece of the Continent, A Part of the Main”: IPRs, Pandemics and the Need for Bottom-Up Approaches for Institutional Change Towards Global Equitable Access to Vaccines?

Arguably, given the challenge of securing affordable global access to vaccines and other health technologies during COVID-19 and in the WHO Pandemic Agreement discussions, there is a broader need for a shift in thinking within the health innovation landscape, including around how IPRs operate and which decision-making actors, aside from rightsholders, can exert control in such contexts. This section will argue that a deeper change of institutional thinking around health innovation is needed, which requires a bottom-up change across a range of actors and entities. Arguably, one key element of this change within the current context is embedding much greater recognition of the range of contributions (and resources) necessary in the successful development and delivery of health technologies, including in pandemic contexts. Such an approach would still recognize the role of rightsholders,^105^ and maintain the incentivizing role of IP in this context in developing such technologies. Nonetheless, alongside this, it aims to recognize the contributions of other actors who contribute critical resources to developing health technologies and in this way challenge and curtail the (often exclusive) private governance role that rightsholders have over IP-protected health technologies. The arguments made do not discount the contributions of rightsholders to the health innovation process; rather, they seek to situate these within the broader range of contributions needed for the development of pandemic (and other health) technologies. More specifically, it argues that a range of other actors (aside from rightsholders) are also key resource providers, and that they should condition access to such resources with clauses that mandate ethical access pathways for the technologies downstream, including in pandemic contexts. Furthermore, this argument aims to situate such contributions within the broader global health context, and the need for IPRs to be used in ways that promote the broader societal good, including the need for global equitable access to health technologies during pandemic contexts.

Aside from original inventors/rightsholders, key actors that provide critical resources for the development of health technologies — often with a core aim of facilitating the development of health technologies in the public good — include but are not limited to the following intermediary bodies.^106^ First, *funders* of research that act as *financial resource providers,* as such funding is used to support research related to the development of a health technology (e.g., a medicine). The term “funders” should be interpreted broadly to include a variety of entities that provide financial support to the development of research underpinning the development of health technologies. For example, various aspects of scientific research are supported by public funding bodies, such as Horizon Europe, national funders, etc. As other commentators have also suggested, such funders could attach conditions to funding that they provide,^107^ including in the form of clauses on how technologies developed downstream are used and developed. In doing so, they could leverage their funding contribution to the research process, conditioning it in a manner that seeks to broaden global access, including during pandemic contexts.

Second, relatedly, alongside the critical role of such types of research funding in underpinning the research needed to develop vaccines and other health technologies, national governments (or regional entities such as the EU) during the COVID-19 pandemic agreed to Advance Purchase Agreements (APA) for vaccines under development. APAs can be used in ways that de-risk companies in developing vaccines and that provide significant investments. Slade and Hawkins’s empirical work examining APAs in the COVID-19 context has highlighted that “consistent across all agreements is the control that the pharmaceutical companies have maintained over these valuable IP rights.”^108^ Slade and Hawkins have argued contractual conditions should be attached to such APAs, and more recently, Anderson, Hawkins and Slade have made a very convincing case for these conditions to be adopted across the translational research and development chain more generally.^109^ Such conditions could include clauses aimed at securing public interests in access to downstream technologies, including around global supply in pandemic contexts.

Third, significant knowledge is needed to develop health technologies including around core components of a technology, which in some cases will be developed in university contexts. Thus, universities (and other entities where research is being developed) could be seen as key *knowledge providers* by facilitating employees to develop knowledge and critical research for the development of health technologies and acting as key resources for the generation of such knowledge, some of which may lead to IPRs. Moreover, such universities could be seen as financial resource providers, as they also support research necessary for the development of health technologies, including by employing scientists, whose contracts will typically include a proportion of time dedicated to research. Accordingly, universities in such contexts may be seen as acting akin to funders by supporting the conduct of such research (if this is conducted outside of a specific grant). Universities could leverage these resources via a range of avenues, including, for example, by attaching conditions: (a) in their employment contracts with employees, which could stipulate how IPRs and knowledge generated in the course of employment are controlled (this is already a standard feature in many contexts); and (b), relatedly, attaching conditions within their IP licensing agreements around downstream uses of IPRs generated in the university context, which could include ethical access licensing clauses that aim to facilitate global equitable access to technologies developed in university context or developed using university IP. Indeed, such clauses on downstream access to technologies developed in university contexts are recommended internationally, such as by the Association of University Technology Transfer Managers’ Nine Points Document (2007).^110^

Fourth, a key resource needed for scientific research is having appropriate biological specimens for health research. Biobanks or tissue repositories provide key *biological resources in such contexts* as they contribute samples for use in health research. Such samples in the case of publicly funded biobanks are often obtained from members of the public who typically donate samples altruistically, for a range of reasons, including with the aim of contributing to the development of new health technologies.^111^ There is scope for biobanks to impose conditions on the use of such samples, including conditions that facilitate access to downstream technologies developed, such as in pandemic contexts.

Arguably, such resource providers’ (often publicly funded entities if we consider biobanks, funders, and universities) and individuals’ (members of the public) contributions to the health innovation context should have greater recognition. These contributions (without which health innovation is not possible in many cases) should be leveraged as far as possible by these intermediary bodies, alongside other mechanisms within IP contexts towards addressing public interests at stake,^112^ including towards ensuring broader equitable access to downstream technologies developed during pandemic contexts. In the short term, this would enable additional tangible pathways to securing access to health technologies in pandemic (and also potentially other) contexts.

Moreover, in the longer term, embedding such an approach across multiple layers within the health innovation system could be used as one key step to shift the institutional culture towards ensuring affordable/equitable access to health technologies. This in turn could then set up clearer foundations to support the implementation of the WHO Pandemic Agreement (and other initiatives) including some of the clauses within it for equitable access to health technologies during pandemics in ways that prioritize global health needs. It could also set up a system that may be more receptive to supporting future multilateral actions around pandemic preparedness in this and other contexts. However, for such an approach to be effective, it is vital that this type of consideration and leveraging of various resources/contributions to the health innovation context in ways that secure better downstream access to such health technologies would become normalized across the system including by funders, biobanks, and universities (and other relevant actors). If collective approaches develop, then more funders, biobanks, universities, etc., are likely to adopt such conditions; otherwise they may be concerned that companies will choose to work with other entities instead, and this may deter them from the adoption of conditions. Ideally, this would happen across jurisdictions, as this would provide greater strength to such developments.

Moreover, the effectiveness of such approaches would also hinge on how any conditions were framed in this context; they would need to be specific enough to provide tangible action on access to health technologies, yet would need to also be broad enough to encompass a range of contexts and scientific developments. Arguably, model clauses should be developed at an international level that engage with views of a range of stakeholders across the health innovation landscape with these aims in mind — however, the precise content of any such clauses is beyond the scope of this article.^113^ Nonetheless, such conditions on the provision of resources are a necessary component within the current system of seeking to mandate a better balance between incentives that may be provided by IPRs and the health implications certain uses of IPRs can have, particularly in pandemic contexts. Having said this, alongside seeking to rebalance rightsholders’ governance role in this way, it is also vital that other mechanisms are implemented for future pandemic preparedness that ensure that the existing IP and technology transfer mechanisms within the adopted WHO Pandemic Agreement will likely be implemented in a manner which contributes towards global equitable access to vaccines for the next pandemic. In this context, a range of mechanisms can and should be considered to maximize voluntary sharing of know-how and technology transfer and develop upon provisions within the WHO Pandemic Agreement. However, alongside this, in case such voluntary mechanisms fail, arguably mandatory mechanisms may also be necessary. For example, in the technology transfer context, Gurgula and McDonagh put forward a proposal for an Article 11 bis to be added to the draft WHO Pandemic Agreement. Their proposal provided for an important non-voluntary technology transfer mechanism that could be used to supplement existing provisions in the Agreement, and enabled such a mechanism to be invoked in pandemic contexts in the event that voluntary technology transfer mechanisms failed. Although the WHO Pandemic Agreement (May 2025) has now been adopted, it is still vital we consider the need for such non-voluntary mechanisms. Hopefully, such mechanisms will not be needed for future pandemics, but in the event they are, such mechanisms are vital to have in place to enable us to avoid issues that arose in the COVID-19 context.^114^ Furthermore, coordination on such initiatives would be needed to ensure the approaches align and work together towards a collective aim of facilitating avenues for global equitable access to vaccines (and other health technologies) in pandemic contexts.

## Part V: Conclusion

Focusing on IPRs in the contexts of access to pandemic health technologies, this article has argued that COVID-19 and some of the developments in negotiations around the WHO Pandemic Agreement (May 2025) have demonstrated an individualistic approach towards the use of IPRs over health technologies in pandemic contexts. It has made the case that arguably such individual modes of thinking on the part of states, regions, and rightsholders are legally and institutionally embedded and may be difficult to shift by solely using top-down approaches such as via the adoption of an international legal instrument. Nonetheless, the fact that a consensus was reached on a pandemic text is a recognition of a commitment to an international response in this context. However, the provisions within the agreed text related to IPRs and how these can be used over pandemic health technologies leave much discretion to states and contrast with the broader ambition of the Zero-text, having been watered down during negotiations. In practice, in terms of the success of this instrument, much will depend on how the provisions within it are implemented in practice over time.

For future pandemic preparedness to better ameliorate the access issues posed by how IPRs are used in such contexts, this article made the case for bottom-up institutional changes that start from a fundamentally different and broader conception of value around the contributions (or resources) necessary for the development of health technologies, including in pandemic contexts. As one key element of this, there is a need for a broader recognition of a range of contributions needed to develop emerging health technologies. This article put forward the case for the need for key resource providers (including publics, biobanks, universities, funders, and states) to better leverage such resources to drive a culture of facilitating affordable equitable access to IPRs over pandemic-related health technologies.

Put simply, the individualized nationalistic and rightsholder-driven approach to the distribution of access to health technologies, as was evident during the COVID-19 pandemic, solidifies rightsholder/market power and contributes towards a de-prioritization of societal public needs and a failure of collective action. This approach does not bode well for pandemic management or preparedness related to IPRs and access to medicines. For future pandemics, a different approach is needed. The WHO Pandemic Agreement is a step that may facilitate this, but much will depend on how it is implemented, and to address issues around global equity and access to health technologies, a much deeper institutional change is needed.
